# Association of intraoperative hypotension with acute kidney injury after liver resection surgery: an observational cohort study

**DOI:** 10.1186/s12882-020-02109-9

**Published:** 2020-11-02

**Authors:** Pingping Liao, Shuo Zhao, Lin Lyu, Xuanlong Yi, Xiangyu Ji, Jian Sun, Yanfang Jia, Zangong Zhou

**Affiliations:** 1 Department of Geriatric Medicine, the Affiliated Hospital of Qingdao University, Qingdao, 266000 Shandong China; 2 Department of Anesthesiology, the Affiliated Hospital of Qingdao University, No.16 Jiangsu Road, Qingdao, 266000 Shandong China; 3 Department of Anesthesiology, the Affiliated Hospital of Qingdao University, No.59 Haier Road, Qingdao, 266000 Shandong China

**Keywords:** Acute kidney injury, Intraoperative hypotension, Liver resection

## Abstract

**Background:**

Acute kidney injury (AKI) is a major complication following liver resection. The aim of this study was to determine the risk factors for AKI after hepatic resection and whether intraoperative hypotension (IOH) was related to AKI.

**Methods:**

Adult patients (≥ 18 years) undergoing liver resection between November 2017 and November 2019 at our hospital were retrospectively reviewed. AKI was defined as ≥50% increase in serum creatinine from baseline value within 48 h after surgery. IOH was defined as the lowest absolute mean arterial pressure (MAP) < 65 mmHg for more than 10 cumulative minutes during the surgery. Patients were divided into AKI group and non-AKI group, and were stratified by age ≥ 65 years.

**Results:**

796 patients who met our inclusion and exclusion criteria were analyzed. After multivariable regression analysis, the IOH (OR, 2.565; *P* = 0.009) and age ≥ 65 years (OR, 2.463; *P* = 0.008) were risk factors for AKI. The IOH (OR, 3.547; *P* = 0.012) and received red blood cell (OR, 3.032; *P* = 0.036) were risk factors of AKI in age ≥ 65 years patients.

**Conclusions:**

The IOH and age ≥ 65 years were risk factors for postoperative AKI, and IOH was associated with AKI in age ≥ 65 years patients following liver resection.

**Supplementary information:**

**Supplementary information** accompanies this paper at 10.1186/s12882-020-02109-9.

## Background

Postoperative acute kidney injury (AKI) is a major complication following liver resection, of which the incidence is reported approximately 8.7–15% [1, 2]. AKI in patients who have undergone liver resection is associated with prolonged hospital stay and hospital mortality [3] . As similar to AKI following other type of surgeries [4], AKI following liver resection also increases the risk of mortality or developing chronic kidney disease (CKD) in the long term [5].

Many factors inducing renal cycles of ischemia and reperfusion during surgery lead to postoperative AKI, including high rates and volumes of blood product transfusion or infused fluid, use of vasopressors and blood loss etc. [6]. It is proposed that intraoperative hypotension (IOH) resulting in disruption of renal perfusion may induce AKI [7]. As revealed by many studies, intraoperative decreased mean arterial pressure (MAP) was associated with postoperative AKI [8, 9].

Liver resection surgery is one of the most challenging abdominal surgical procedures. Characteristics of liver resection including clamping of portal vein, massive bleeding and low central venous pressure (CVP) technique induce low MAP during surgery [10–12]. Several studies have identified risk factors for postoperative AKI [3, 13]. However, the association between IOH and postoperative AKI in liver resection surgery was unknown. Therefore, we conducted a single-center retrospective study to determine the risk factors for AKI after hepatic resection surgery and whether IOH was related to postoperative AKI.

## Methods

### Study design and population

This retrospective study was exempted from formal institutional review board of our hospital because these was no modified intervention or disclosure of personal information and the informed consent from patients was waived.

Adult patients (≥ 18 years) undergoing liver resection between November 2017 and November 2019 at Affiliated Hospital of Qingdao University were retrospectively reviewed. The following procedures were excluded: pediatric patients (< 18 years), patients with kidney disease or kidney surgery, liver resection combined with other surgery, liver radiofrequency ablation, liver resection for liver donation, nonmajor liver procedures (eg, biopsy and palliative operation), second surgery, post liver transplantation, trauma, metastases and patients without preoperative or postoperative creatinine.

### Data collection, definition and outcomes

AKI was defined as ≥50% increase in serum creatinine (sCr) from baseline value (preoperative) within 48 h after surgery based on Acute Kidney Injury Network (AKIN) creatinine criteria [14]. Urine output was not available in our postoperative cohort, so the AKIN urine output criteria was not used in this study. Demographics of patients, surgical and anesthetic information, laboratory values and postoperative data were manually abstracted from the electronic medical record database. Demographics of patients included: age, sex, body weight, American Society of Anesthesiologists (ASA) physical status, history of hypertension, diabetes, coronary heart disease (CHD), drugs use [ACEI/ARB (angiotensin converting enzyme inhibitors/angiotensin receptor blockers), NSAIDs (non-steroidal anti-inflammatory drugs), diuretics] and hepatitis B. The surgical and anesthetic information were: pre-operative MAP, laparoscope or not, major or minor resection, surgery duration, use of vasoactive inotropic agent, blood loss, urine volume, transfusion, nadir MAP, IOH or not, clamping of portal vein times and clamping of portal vein duration. The type of liver resection was classified as either minor resection (partial hepatectomy) or major resection (right, left, or trisectionsectomy). The MAP values were not directly measured, MAP was calculated as (1/3 × systolic blood pressure + 2/3 × diastolic blood pressure). Preinduction baseline MAP was obtained as the first valid value upon arrival to the operating room on the date of surgery as previous study defined [8]. IOH was defined as the lowest absolute MAP < 65 mmHg for more than 10 cumulative minutes during the surgery [7]. The baseline sCr was defined as the preoperative sCr collected closest to the date of surgery within 1 month before surgery. The eGFR (estimated glomerular filtration rate) was calculated by Cockcroft-Gault Equation: eGFR (ml/min/1.73 m2) = creatinine clearance (Ccr) × 0.84 × 1.73/body surface are (BSA); Ccr = [(140-age) × weight×(0.85 if female)]/(72 × sCr). Postoperative sCr was the max sCr within 48 h after surgery, and postoperative aspartate aminotransferase (AST) and alanine aminotransferase (ALT) was abstracted the max value 7 days after surgery.

Intensive care unit (ICU) admission, hospital length of stay, re-operation and mortality were included in this study. Re-operation was defined as exploratory laparotomy because of biliary leakage and/or the presence of bleeding. Mortality was defined as any death occurring within 30 days following the date of surgery or in-hospital mortality.

### Statistical analysis

All statistical analyses were performed using SPSS software (IBM Corp. Released 2012. IBM SPSS Statistics for Windows, Version 21.0. Armonk, NY: IBM Corp.). Categorical variables were expressed as frequencies with percentages and compared with chi-square test or Fisher’s exact test as appropriate. Continuous variables were presented as mean ± standard deviation or median with 25th to 75th percentiles, and were compared using the Student’s t-test or Mann-Whitney-U test, where appropriate. Variables with a *p* < 0.1 in univariate analysis or variables deemed important for clinical outcomes were further analyzed in multivariable regression model. Logistic regression analysis was used to identify risk factors associated with AKI after liver resection surgery. According to the result of logistic regression, age ≥ 65 years and intraoperative hypotension were risk factors to AKI after liver resection. Therefore, we stratified the patients into age < 65 years group and age ≥ 65 years group according to other studies about elderly AKI [15–17]. Statistical significance was considered as a two-tailed *p* < 0.05.

## Results

Of 845 patients having liver surgery between November 2017 and November 2019 at Affiliated Hospital of Qingdao University, analysis included 796 patients who met our inclusion and exclusion criteria. The average age was 59 [52–65] years and male/female was 484/312. The rate of age ≥ 65 years patients was 29.1%. Indications for surgery were: hepatocellular carcinoma in 574 patients (72.1%), cholangiocarcinoma in 115 patients (14.4%), hepatolithiasis in 89 patients (11.2%), hepatic hemangioma in 11 patients (1.4%) and gallbladder cancer in 7 patients (0.9%). The overall incidence of AKI was 5.0% [AKIN1, 31(3.9%); AKIN2, 6(0.7%); AKIN3, 3(0.4%)], and the incidence of AKI in age ≥ 65 years group was 9.1%. None of AKI patients needed continuous renal replacement therapy. The mortality was 0.4%.

The demographics of patients between AKI and Non-AKI group are shown in Table 1. Patients with AKI was older than patients without AKI (*p* = 0.003), and the rate of age ≥ 65 years patients was higher in AKI group (*p* = 0.002). AST and ALT were higher in AKI group. Baseline sCr (*p* = 0.056) and eGFR (*p* = 0.540) were not different between AKI group and Non-AKI group. The intraoperative characteristics and outcomes are presented in Table 2 between Non-AKI and AKI group. The intraoperative nadir MAP was lower in AKI group (*p* = 0.027), and the incidence of IOH was higher in AKI group during surgery (*p* < 0.001). The rate of norepinephrine use and the rate of received red blood cell and received plasma were high in AKI patients. The patients developed AKI suffered longer surgery duration (*p* = 0.001) and more blood loss (*p* = 0.011) and needed more volumes of infused crystalloid (*p* = 0.006). The AKI group was associated with higher rate of ICU admission and longer hospital length of stay compared with the non-AKI group. As the result of univariate analysis, AST before surgery, IOH, rate of age ≥ 65 years, norepinephrine use, received red blood cell, received plasma, volumes of infused crystalloid, blood loss, urine volume and surgery duration were entered into multiple logistic regression analysis. The IOH and age ≥ 65 years were risk factors for AKI after liver resection (Table 4).

**Table 1:** Demographics of patients between Non-AKI group and AKI group

Parameter	Non-AKI group (*n* = 756)	AKI group (*n* = 40)	P	Chi-squared
Age (years), Median [25th to 75th P]	59 [51–65]	65 [56–68]	0.003	
≥65 years, n(%)	211 (27.9%)	21 (52.5%)	0.001	11.124
Weight (kg), Median [25th to 75th P]	66.0 [60.0–75.0]	68.5 [56.5–73.5]	0.664	
Height (cm), Median [25th to 75th P]	167.0 [160.0–172.0]	166.0 [158.8–172.3]	0.417	
Male, n(%)	460 (60.8%)	24 (60.0%)	0.915	0.011
Hypertension, n(%)	176 (23.3%)	14 (35.0%)	0.090	2.871
Diabetes, n(%)	102 (13.5%)	9 (22.5%)	0.109	2.569
CHD, n(%)	43 (5.7%)	5 (12.5%)	0.086	
Hepatitis B, n(%)	307 (40.6%)	12 (30.0%)	0.182	1.780
ACEI/ARB, n(%)	56 (7.4%)	6 (15.0%)	0.118	
Diuretics, n(%)	1 (0.13%)	0 (0%)	1.000	
NSAIDs, n(%)	24 (3.2%)	2 (5.0%)	0.380	
ASA class1–2/class 3–4	406/350	16/24	0.091	2.864
Major/Minor resection	230/526	14/26	0.541	0.374
Laparoscope, n(%)	440	21	0.477	0.507
Preinduction baseline MAP (mmHg), Median [25th to 75th P]	96.7 [90.0–106.7]	98.3 [93.3–105.4]	0.351	
Basic laboratory characteristics				
AST (IU/L), Median [25th to 75th P]	24 [18–34]	28 [21–47]	0.018	
ALT (IU/L), Median [25th to 75th P]	24 [16–41]	30 [20–50]	0.041	
sCr (μmol/L), Median [25th to 75th P]	64.0 [53.0–77.7]	55.0 [47.6–74.0]	0.056	
eGFR (ml/min/1.73m^2^), Median [25th to 75th P]	82.2 [67.3–100.4]	87.9 [68.5–105.1]	0.540	
BUN (μmol/L), Median [25th to 75th P]	5.4 [6.3–4.3]	5.4 [3.8–6.2]	0.438	

*AKI* acute kidney injury, *25th to 75th P* 25th to 75th percentiles, *CHD* coronary heart disease, *ACEI* angiotensin converting enzyme inhibitors, *ARB* angiotensin receptor blockers, *NSAIDs* non-steroidal anti-inflammatory drugs, *ASA* american society of anesthesiologists, *MAP* mean arterial pressure, *AST* aspartate aminotransferase, *ALT* alanine aminotransferase, *sCr* serum creatinine, *eGFR* estimated glomerular filtration rate, *BUN* blood urea nitrogen

**Table 2:** Intraoperative characteristics and outcomes between Non-AKI group and AKI group

Parameter	Non-AKI group (*n =* 756)	AKI group (*n =* 40)	P	Chi-squared
Intraoperative characteristics				
Nadir MAP (mmHg), mean ± SD	71.52 ± 8.19	66.92 ± 12.33	0.027	
Hypotension, n(%)	145 (19.2%)	19 (47.5%)	< 0.001	18.626
Norepinephrine use, n(%)	72 (9.5%)	9 (22.5%)	0.015	
Dopamine use, n(%)	16 (2.1%)	1 (2.5%)	0.588	
Milrinone use, n(%)	91 (12.0%)	6 (15.0%)	0.617	
Received red blood cell, n(%)	76 (10.1%)	11 (27.5%)	0.002	
Received plasma, n(%)	45 (6.0%)	9 (22.5%)	0.001	
Crystalloid (ml), Median [25th to 75th P]	1500 [1000–1500]	1500 [1000–2000]	0.006	
Hydroxyethyl starch (ml), Median [25th to 75th P]	500 [500–500]	500 [500–1000]]	0.211	
Blood loss (ml), Median [25th to 75th P]	200 [100–300]	300 [100–775]	0.011	
Urine volume (ml), Median [25th to 75th P]	400 [200–500]	500 [263–700]	0.051	
Clamping of portal vein times (n), Median [25th to 75th P]	1 [1–2]	2 [1–2]	0.609	
Clamping of portal vein duration (min), Median [25th to 75th P]	20 [15–30]	25 [17–30]	0.736	
Surgery duration (hours), Median [25th to 75th P]	3.0 [2.5–4.0]	4.0 [3.1–4.5]	0.001	
Postoperative laboratory characteristics				
Peak AST (IU/L), Median [25th to 75th P]	172.9 [101.9–313.0]	303.7 [151.5–721.0]	0.001	
Peak ALT (IU/L), Median [25th to 75th P]	182 [106–337]	269 [150–715]	0.002	
Peak sCr (μmol/L), Median [25th to 75th P]	64.0 [53.0–76.1]	91.5 [74.4–138.5]	< 0.001	
Minimum eGFR (ml/min/1.73m^2^), Median [25th to 75th P]	81.2 [68.1–100.6]	53.1 [38.4–69.7]	< 0.001	
Peak BUN (μmol/L), Median [25th to 75th P]	5.7 [4.8–6.8]	7.3 [5.5–10.0]	< 0.001	
ICU admission, n(%)	46 (6.1%)	7 (17.5%)	0.013	
Hospital length of stay (days), Median [25th to 75th P]	12 [10–14]	14 [10–20]	0.027	
Re-operation, n(%)	4 (0.5%)	1 (2.5%)	0.228	
Mortality, n(%)	2 (0.3%)	1 (2.5%)	0.144	

*AKI* acute kidney injury, *MAP* mean arterial pressure, *25th to 75th P* 25th to 75th percentiles, *AST* aspartate aminotransferase, *ALT* alanine aminotransferase, *sCr* serum creatinine, *eGFR* estimated glomerular filtration rate, *BUN* blood urea nitrogen, *ICU* intensive care unit

In order to investigate whether elderly patients are sensitive to IOH for suffering AKI, we stratified the patients into age < 65 years group and age ≥ 65 years group. The rate of ASA class 3–4 was higher in AKI group in age < 65 years patients (*p* = 0.032), and other characteristics before surgery was not different between AKI group and Non-AKI group in age < 65 years patients (supplementary Table S1). There was no difference in preoperative characteristics between AKI group and Non-AKI group in age ≥ 65 years patients. AKI group suffered longer surgery duration compared with non-AKI group in age < 65 years patients (*p* = 0.033). Although the rate of IOH was higher in AKI group compared with non-AKI group in age < 65 years patients, there was no statistical difference between two groups (*p* = 0.070, Table 3). The rate of IOH was higher in AKI group compared with non-AKI group in age ≥ 65 years patients (*p* = 0.001, Table 3). The rate of norepinephrine use (*p* = 0.008), the rate of received red blood cell (*p =* 0.001) and the rate of received plasma (*p =* 0.001) were higher in AKI group in age ≥ 65 years patients. The patients developed AKI suffered longer surgery duration (*p* = 0.030) and more blood loss (*p* = 0.016) in age ≥ 65 years patients. The AKI group was associated with higher rate of ICU admission (*p* = 0.004) and longer hospital length of stay (*p* = 0.028) compared with the non-AKI group in age ≥ 65 years patients. According to the results of univariate analysis, ASA class, ALT before surgery, IOH, volumes of infused crystalloid and surgery duration were entered into logistic regression analysis. ASA class 3–4 was a risk factor for AKI in age < 65 years patients (Table 4). The AST before surgery, IOH, norepinephrine use, volumes of infused crystalloid, the rate of received red blood cell and received plasma, blood loss and urine volume were further analyzed by multivariable logistic regression. The IOH (*p* = 0.012) and received red blood cell (*p* = 0.036) were associated with AKI in age ≥ 65 years patients (Table 4).

**Table 3:** Intraoperative characteristics and outcomes between Non-AKI group and AKI group stratified by age ≥ 65 years

	Age < 65 years				Age ≥ 65 years			
	Non-AKI group (*n* = 545)	AKI group (*n* = 19)	P	Chi-squared	Non-AKI group (*n* = 211)	AKI group (*n =* 21)	P	Chi-squared
Intraoperative characteristics								
Nadir MAP (mmHg), mean ± SD	71.83 ± 8.24	69.65 ± 12.41	0.469		70.72 ± 8.02	64.59 ± 12.06	0.033	
Hypotension, n(%)	102 (18.7%)	7 (36.8%)	0.070		43 (20.4%)	12 (57.1%)	0.001	
Norepinephrine use, n(%)	50 (9.2%)	2 (10.5%)	0.692		22 (10.4%)	7 (33.3%)	0.008	
Dopamine use, n(%)	14 (2.6%)	0 (0)	1.000		2 (0.9%)	1 (4.8%)	0.249	
Milrinone use, n(%)	56 (10.3%)	3 (15.8%)	0.437		35 (16.6%)	3 (14.3%)	1.000	
Received red blood cell, n(%)	49 (9.0%)	2 (10.5%)	0.686		27 (12.8%)	9 (42.9%)	0.001	
Received plasma, n(%)	30 (5.5%)	2 (10.5%)	0.294		15 (7.1%)	7 (33.3%)	0.001	
Crystalloid (ml), Median [25th to 75th P]	1400 [1000–1500]	1500 [1000–2000]	0.057		1500 [1000–1500]	1500 [1000–2325]	0.061	
Hydroxyethyl starch (ml), Median [25th to 75th P]	500 [500–500]	500 [500–500]	0.950		500 [500–563]	500 [500–1000]	0.119	
Blood loss (ml), Median [25th to 75th P]	200 [100–300]	200 [50–400]	0.357		200 [100–325]	500 [150–1150]]	0.016	
Urine volume (ml), Median [25th to 75th P]	300 [200–500]	400 [250–600]	0.468		400 [200–600]	500 [250–800]	0.068	
Clamping of portal vein times (n), Median [25th to 75th P]	1 [1–2]	1.5 [1–2]	0.933		1 [1–2]	2 [1–2.5]	0.297	
Clamping of portal vein duration (min), Median [25th to 75th P]	25 [15–30]	25 [16–29]	0.959		20 [15–30]	20 [17–43]	0.586	
Surgery duration (hours), Median [25th to 75th P]	3.0 [2.5–4.0]	4.0 [3.0–4.5]	0.033		3.5 [2.5–4.5]	4.0 [3.3–5.0]	0.030	
Postoperative laboratory characteristics								
Peak AST (IU/L), Median [25th to 75th P]	174 [101–331]	310 [162–718]	0.066		164 [103–264]	298 [140–800]	0.005	
Peak ALT (IU/L), Median [25th to 75th P]	192 [113–371]	272 [181–600]	0.031		155 [96–282]	266 [128–1052]	0.008	
Peak sCr (μmol/L), Median [25th to 75th P]	62.4 [51.7–75.0]	90.0 [67.2–141.0]	< 0.001		66.0 [56–80.0]	95.0 [74.8–136.5]	< 0.001	
Minimum eGFR (ml/min/1.73m^2^), Median [25th to 75th P] or mean ± SD	89.7 [74.5–108.1]	62.3 [42.2–72.4]	< 0.001		68.67 ± 17.66	46.59 ± 18.08	< 0.001	
Peak BUN (μmol/L), Median [25th to 75th P]	5.5 [4.6–6.5]	6.3 [5.2–8.8]	0.009		6.4 [5.3–7.5]	7.4 [5.6–13.1]	0.029	
ICU admission, n(%)	27 (5.0%)	0 (0)	1.000		19 (9.0%)	7 (33.3%)	0.004	
Hospital length of stay (days), Median [25th to 75th P]	11 [9–14]	13 [9–15]	0.505		13 [10–15]	16 [11–26]	0.028	
Re-operation, n(%)	3 (0.6%)	1 (5.3%)	0.128		1 (0.5%)	0 (0)	1.000	
Mortality, n(%)	1 (0.2%)	0 (0)	1.000		1 (0.5%)	1 (4.8%)	0.173	

*AKI* acute kidney injury, *MAP* mean arterial pressure, *SD* standard deviation, *25th to 75th P* 25th to 75th percentiles, *AST* aspartate aminotransferase, *ALT* alanine aminotransferase, *sCr* serum creatinine, *eGFR* estimated glomerular filtration rate, *BUN* blood urea nitrogen, *ICU* intensive care unit

**Table 4:** Logistic regression model for risk factors of AKI

Risk factors	β	SE	Odds ratio	95%CI	P
IOH	0.942	0.358	2.565	1.271–5.177	0.009
Age ≥ 65 years	0.901	0.340	2.463	1.264–4.800	0.008
	Age < 65 years				
ASA class 3–4	−1.251	0.630	0.372	0.083–0.983	0.047
	Age ≥ 65 years				
IOH	1.266	0.503	3.547	1.323–9.505	0.012
Received red blood cell	1.109	0.530	3.032	1.073–8.568	0.036

*AKI* acute kidney injury, *IOH* intraoperative hypotension, *ASA* American Society of Anesthesiologists, *SE* standard error, *CI* confidence interval

## Discussion

In this single-center cohort study, the total incidence of AKI after liver resection was 5.0%, and the incidence of AKI in age ≥ 65 years group was 9.1%. The incidence of AKI is lower than results reported by other studies [1, 2]. The reason of low occurrence of AKI in our cohort is that the liver resection combined with other surgery, second surgery, post liver transplantation and metastases were excluded in this study. So the surgery in our study is less complicated than other studies [15, 18].

We found that the IOH was a risk factor for AKI after liver resection. Various definitions of intraoperative low MAP (65–55 mmHg) which is related to AKI have been evaluated in the literature [8, 9]. The incidence of AKI increased with the decrease of MAP during surgery [8]. Therefore, we chose upper limit (absolute MAP < 65 mmHg during the surgery) as the threshold in this study [7]. Rhee et al. measured the renovascular reactivity to low MAP by a piglets experiment [19]. This study showed that renal blood flow decreased to 75, 50, and 25% of baseline when MAP decreased to 60, 45, and 40 mmHg. Blood loss remains a major complication in liver resection surgery [10], which is the main reason of low MAP during surgery. AKI patients suffered more blood loss in our study, which is consistent with other study [20]. To avoid hypotension during surgery, blood transfusion, volumes of infused fluid and frequency use of norepinephrine were employed to maintain an acceptable level of MAP. These techniques were used more in AKI group in this study. The intraoperative red blood cell transfusion has been proved to be a risk factor of AKI after liver resection surgery [3]. Many biochemical and biomechanical changes associated with erythrocytes storage include depletion of ATP and 2,3-DPG, membrane phospholipid vessiculation and loss, lipid peroxidation of red blood cell membrane. These changes cause impaired oxygen unloading from hemoglobin and loss of deformability. The loss of deformability leads to obstructed capillaries and then causes impairment of oxygen delivery to tissues [21]. Use of norepinephrine may potentially leads to decrease in renal blood flow and renal oxygen delivery. This result has been proved by animal experiments and healthy volunteers [22, 23]. However, restoration of MAP from 60 to 75 mmHg induced by norepinephrine increases renal oxygen delivery, glomerular filtration rate and renal oxygenation in post-cardiac surgery AKI patients [24]. Therefore, low MAP corrected by norepinephrine when MAP is below 60 mmHg may improve renal perfusion in liver resection surgery.

In this study, mean age was higher in AKI group, and the incidence of age ≥ 65 years was identified as a risk factor for AKI following liver resection. Although Dedinska et al., [15] found that the incidence of AKI was higher in elderly patients following liver resection surgery, the age was not an independent risk factor for AKI. This may be explained by the different inclusion. In the Dedinska et al., [15] study, colorectal carcinoma with metastases in the liver were more frequent in the group of patients older than 65 years and the histological identification of colorectal carcinoma metastases was a risk factor for AKI. Therefore, the patients older than 65 years may be a risk factor when colorectal carcinoma with metastases was excluded. It has been suggested in a previous study that glomerular filtration rate and autoregulation of renal blood flow decrease with the ageing [25]. Hence this physiologic decline in renal function may predispose the elderly patients to the subsequent development of AKI. In addition, elderly patients often have comorbidities before surgery such as hypertension, cardiovascular disease and diabetes mellitus. These comorbidities make elderly patients susceptible to AKI [26].

In order to investigate the relationship between IOH and AKI in elderly patients, patients were stratified into age < 65 years group and age ≥ 65 years group. We found the IOH was a risk factor of AKI in elderly patients, while IOH was not a risk factor for AKI in patients with age < 65 years. It is more difficult to manage perioperative hemodynamic instability in elderly patients than that in young patients [27]. The GFR (glomerular filtration rate) was associated with left ventricular function and the elderly patients with impaired left ventricular function are susceptive to AKI [28]. In addition, the aging kidney diminishes responsiveness to vasodilators and increases sensitivity to vasoconstrictors [29], which enhance renal ischemia. Fluid therapy is employed to correct hypotension during surgery. However, fluid overload increases burden on elderly renal causing by decreased number of glomerular and tubular volume in aging renal [30], and fluid overload increases AKI severity [31]. A recent review suggested that the haemodynamic management of the elderly patients with surgery should focus on avoiding hypotension and high central venous pressures (CVP) during surgery to decrease risk of AKI [32]. In this study, the rate of received red blood cell and received plasma were higher in elderly patients with AKI. Several animal experiments have shown that aging rats are more susceptible to renal ischemia-reperfusion injury [33, 34]. A sudden substantial blood loss is often associated with a period of decreased MAP during surgery, which causes renal hypoperfusion. Meanwhile, the treatment with overload fluid infusion, blood transfusion and vasopressors to elevate MAP results in renal injury in elderly patients. That may explain that IOH is a risk factor of AKI in elderly patients. Therefore, patients especially elderly patients with surgery need perioperative hemodynamic optimization to decrease risk of renal impairment [35].

Our results are broadly consistent with the results of previous studies that postoperative AKI is associated with significantly increased rates of ICU admission and hospital length of stay in patients with liver surgery [3, 15]. Other studies have reported that short-term mortality is higher in AKI patients after liver resection [18].

There are several limitations to the present study. First, this was a retrospective single center study, so it had the inherent potential for bias. Second, the effect of low CVP strategy or persistent high level of CVP on the occurrence of AKI was not evaluated, since few CVP was continuously monitored and recorded during the surgery. Third, the IOH was not stratified by intraoperative MAP value and low MAP duration because of small sample size. Finally, whether IOH is associated with CKD in AKI patients after liver resection surgery should be studied by further research.

## Conclusions

IOH and age ≥ 65 years were risk factors for AKI after liver resection, and IOH was associated with AKI in age ≥ 65 years patients following liver resection.

## Supplementary information

**Additional file 1: Supplementary Table**. Table S1. Demographics of patients between Non-AKI group and AKI group stratified by age ≥ 65 years. AKI, acute kidney injury; 25th to 75th P, 25th to 75th percentiles; SD, standard deviation; CHD, coronary heart disease; ACEI, angiotensin converting enzyme inhibitors; ARB, angiotensin receptor blockers; NSAIDs, non-steroidal anti-inflammatory drugs; ASA, american society of anesthesiologists; MAP, mean arterial pressure; AST, aspartate aminotransferase; ALT, alanine aminotransferase; sCr, serum creatinine; eGFR, estimated glomerular filtration rate; BUN, blood urea nitrogen

## Data Availability

The datasets used and analysed during the current study are available from the corresponding author on reasonable request.

## Abbreviations

AKI: Acute kidney injury
CKD: Chronic kidney disease
IOH: Intraoperative hypotension
MAP: Mean arterial pressure
CVP: Central venous pressure
sCr: Serum creatinine
AKIN: Acute Kidney Injury Network
ASA: American Society of Anesthesiologists
CHD: Coronary heart disease
AST: Aspartate aminotransferase
ALT: Alanine aminotransferase
ICU: Intensive care unit
ACEI: Angiotensin converting enzyme inhibitors
ARB: Angiotensin receptor blockers
NSAIDs: Non-steroidal anti-inflammatory drugs
eGFR: Estimated glomerular filtration rate
