# Supplementary material for: Association of intraoperative hypotension with acute kidney injury after liver resection surgery: an observational cohort study
Source: BMC Nephrol. 2020 Nov 2;21:456. doi: 10.1186/s12882-020-02109-9 (PMC7607844; doi:10.1186/s12882-020-02109-9)
Supplement: Supplementary file 1 — Additional file 1: Supplementary Table. Table S1. Demographics of patients between Non-AKI group and AKI group stratified by age ≥ 65 years. AKI, acute kidney injury; 25th to 75th P, 25th to 75th percentiles; SD, standard deviation; CHD, coronary heart disease; ACEI, angiotensin converting enzyme inhibitors; ARB, angiotensin receptor blockers; NSAIDs, non-steroidal anti-inflammatory drugs; ASA, american society of anesthesiologists; MAP, mean arterial pressure; AST, aspartate aminotransferase; ALT, alanine aminotransferase; sCr, serum creatinine; eGFR, estimated glomerular filtration rate; BUN, blood urea nitrogen [file 12882_2020_2109_MOESM1_ESM.docx]

**Supplementary Table**

Table S1. Demographics of patients between Non-AKI group and AKI group stratified by age ≥ 65 years

|  | Age < 65 years | | | | Age ≥ 65 years | | | |
| --- | --- | --- | --- | --- | --- | --- | --- | --- |
|  | Non-AKI group (n=545) | AKI group (n=19) | P | Chi-squared | Non-AKI group (n=211) | AKI group (n=21) | P | Chi-squared |
| Age (years), Median [25th to 75th P ] | 55 [48-60] | 56 [53-61] | 0.249 |  | 69.0 [66.0-72.0] | 68.0 [66.5-69.5] | 0.356 |  |
| Weight (kg), Median [25th to 75th P ] or mean ± SD | 67 [60-75] | 70 [60-75] | 0.607 |  | 65.61±11.92 | 64.38±10.60 | 0.649 |  |
| Height (cm), Median [25th to 75th P ] | 168 [160-172] | 170 [160-175] | 0.469 |  | 165 [160-170] | 160 [155-170] | 0.121 |  |
| Male, n(%) | 325 (59.6%) | 12 (63.2%) | 0.758 | 0.095 | 135 (64.0%) | 12 (57.1%) | 0.535 | 0.385 |
| Hypertension, n(%) | 100 (18.3%) | 5 (26.3%) | 0.372 |  | 76 (36.0%) | 9 (42.9%) | 0.535 | 0.385 |
| Diabetes, n(%) | 64 (11.7%) | 4 (21.1%) | 0.269 |  | 38 (18.0%) | 5 (23.8%) | 0.556 |  |
| CHD, n(%) | 19 (3.5%) | 2 (10.5%) | 0.154 |  | 24 (11.4%) | 3 (14.3%) | 0.719 |  |
| Hepatitis B, n(%) | 242 (44.4%) | 9 (47.4%) | 0.798 | 0.065 | 65 (30.8%) | 3 (14.3%) | 0.113 | 2.516 |
| ACEI/ARB, n(%) | 35 (6.4%) | 3 (15.8%) | 0.129 |  | 21 (10.0%) | 3 (14.3%) | 0.463 |  |
| Diuretics, n(%) | 0 (0) | 0 (0) |  |  | 1 (0.5%) | 0 (0) | 1.000 |  |
| NSAIDs, n(%) | 15 (2.8%) | 2 (10.5%) | 0.108 |  | 9 (4.3%) | 0 (0) | 1.000 |  |
| ASA class1-2/class 3-4 | 334/211 | 7/12 | 0.032 | 4.588 | 72/139 | 9/12 | 0.423 | 0.641 |
| Major/Minor resection | 164/381 | 7/12 | 0.529 | 0.396 | 66/145 | 7/14 | 0.847 | 0.037 |
| Laparoscope, n(%) | 332 (60.9%) | 11 (57.9%) | 0.791 | 0.070 | 108 (51.2%) | 10 (47.6%) | 0.755 | 0.097 |
| Preinduction baseline MAP (mmHg), Median [25th to 75th P ] | 96.7 [90.0-105.0] | 99.2 [94.6-103.0] | 0.386 |  | 99.47±11.91 | 100.50±11.75 | 0.712 |  |
| Basic laboratory characteristics |  |  |  |  |  |  |  |  |
| AST (IU/L), Median [25th to 75th P ] | 24 [18-35] | 28 [22-45] | 0.111 |  | 24 [18-32] | 27 [21-51] | 0.054 |  |
| ALT (IU/L), Median [25th to 75th P ] | 26 [18-43] | 34 [30-51] | 0.061 |  | 21.3 [14.0-33.5] | 26.0 [19.5-61.5] | 0.075 |  |
| sCr (μmol/L), mean ± SD | 64.47±15.97 | 63.97±24.18 | 0.930 |  | 68.90±17.59 | 61.57±21.45 | 0.076 |  |
| eGFR (ml/min/1.73m^2^), Median [25th to 75th P ] or mean ± SD | 89.8 [73.2-107.6] | 91.1[82.1-112.5] | 0.482 |  | 69.16±17.44 | 79.54±21.34 | 0.042 |  |
| BUN (μmol/L), Median [25th to 75th P ] or mean ± SD | 5.3 [4.2-6.3] | 5.5 [3.8-5.7] | 0.654 |  | 5.67±1.68 | 5.33±1.85 | 0.396 |  |

AKI, acute kidney injury; 25th to 75th P, 25th to 75th percentiles; SD, standard deviation; CHD, coronary heart disease; ACEI, angiotensin converting enzyme inhibitors; ARB, angiotensin receptor blockers; NSAIDs, non-steroidal anti-inflammatory drugs; ASA, american society of anesthesiologists; MAP, mean arterial pressure; AST, aspartate aminotransferase; ALT, alanine aminotransferase; sCr, serum creatinine; eGFR, estimated glomerular filtration rate; BUN, blood urea nitrogen
